# Dermoscopy of neoplastic alopecia secondary to cutaneous metastasis from breast carcinoma^☆^

**DOI:** 10.1016/j.abd.2023.01.009

**Published:** 2024-06-08

**Authors:** Bruno Simão dos Santos, Eduardo César Diniz Macêdo, Bruna Nascimento Arruda Scabello, Patrícia Porto de Oliveira Grossi, Neusa Yuriko Sakai Valente

**Affiliations:** Department of Dermatology, Hospital do Servidor Público Estadual, São Paulo, SP, Brazil

Dear Editor,

This report describes a 68-year-old female patient who presented with a history of asymptomatic skin lesions on the scalp, associated with hair loss, over a four-month period. Six years ago, she had been diagnosed with adenocarcinoma, luminal A subtype, in the left breast, stage pT3N2, and had undergone a mastectomy, adjuvant chemotherapy and radiotherapy, in addition to receiving tamoxifen and anastrozole. Two months before the onset of the dermatological lesions, the patient had been diagnosed with lymph node metastasis from breast carcinoma in the left supraclavicular region.

On dermatological examination of the scalp, three rounded, circumscribed, smooth-surfaced erythematous plaques were observed. These plaques were hardened upon palpation and lacked hair. Two of the plaques, each measuring 2 cm in diameter, were located on the right parietal region and the vertex. The third plaque, measuring 3 cm in diameter, was located on the frontal region (Fig. 1).

**Fig. 1:**
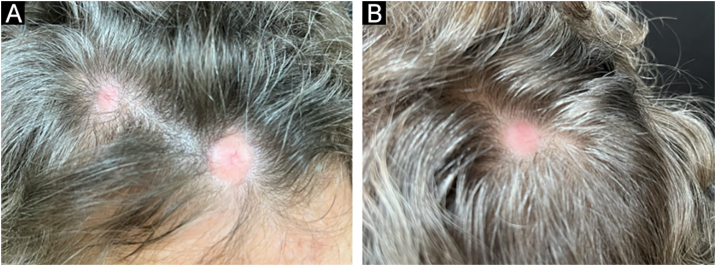
Rounded erythematous plaques of alopecia on the frontal and right parietal regions (A) and on the vertex of the scalp (B).

On dermoscopic examination, the lesions showed a milky-red area, arboriform vessels, fine telangiectasias, shiny white structures, yellow dots, and orange amorphous areas (Fig. 2, Fig. 3).

**Fig. 2:**
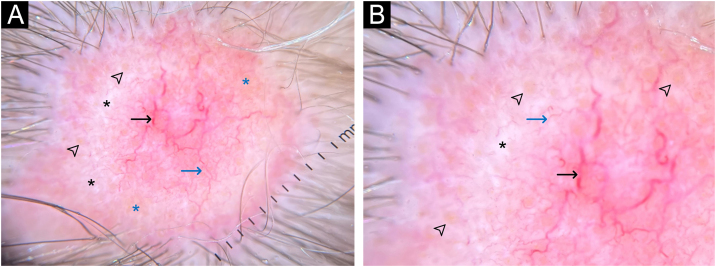
Dermoscopy of the lesion on the frontal region (a and b). Milky-red areas, arboriform vessels (black arrows), fine telangiectasias (blue arrows), shiny white structures (black asterisks), yellow dots (arrowheads), poorly defined orange areas (blue asterisks). Polarized light with contact and immersion fluid (×10).

**Fig. 3:**
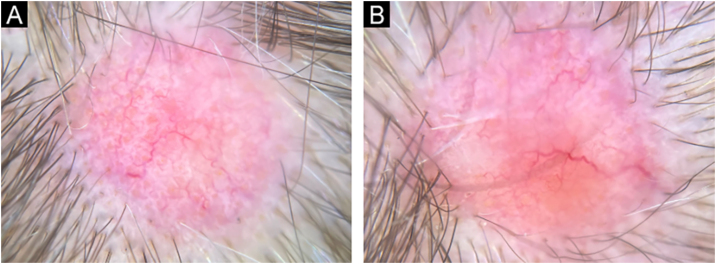
Dermoscopy of lesions on the right parietal region (A) and vertex (B). Dermoscopic pattern similar to that found in the frontal region (Fig. 2, Fig. 3). Polarized light without contact (×10).

An incisional biopsy of the lesion on the frontal region of the scalp showed on histopathology, infiltration of the dermis by atypical epithelial cells arranged in cords with glandular lumens (Fig. 4). Immunohistochemistry was positive for estrogen receptor, GATA 3 and cytokeratins (AE1/AE3) and negative for estrogen receptor. These findings were compatible with cutaneous metastasis from breast carcinoma.

**Fig. 4:**
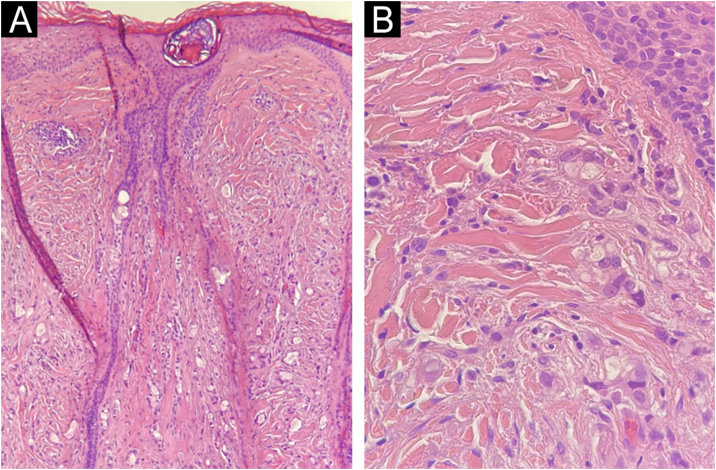
Histopathology of alopecic metastasis in the frontal region. (A) Diffuse infiltration of neoplastic cells in the dermis. Presence of a hair follicle with infundibular dilatation. (B) Detail of the neoplastic infiltration of: atypical epithelial cells, isolated or in small clusters surrounding glandular lumens. Hematoxylin & eosin, ×100 (A) and ×400 (B).

Neoplastic alopecia (NA) is divided into primary, when the neoplasia originates on the scalp, and secondary, when resulting from metastasis.^1^ The most common clinical presentation is cicatricial alopecia, with asymptomatic papules or nodules, erythematous or normochromic, often located on the parietal and frontal regions of the scalp.^2^

In general, the neoplastic cells destroy hair follicles, inducing the recruitment of inflammatory cells and fibroplasia in well-established lesions, which lead to cicatricial alopecia. The main neoplasm associated with NA is breast carcinoma. Other tumors associated with primary or secondary NA are squamous cell carcinoma, basal cell carcinoma, angiosarcoma, gastric adenocarcinoma, placental trophoblastic tumor, and mycosis fungoides.^3^, ^4^

The description of NA and its dermoscopic findings is scarce in the literature. Vezzoni et al. also described a case of NA secondary to malignant neoplasia of the breast, with extensive well-focused arboriform vessels and smaller telangiectasias over a milky-red area and a well-defined orange area, with polymorphic vessels surrounded by yellowish-white scales.^5^ The report by Çetinarslan et al. describes a case of NA secondary to sarcomatoid renal carcinoma, characterized by the presence of yellowish scales in the center of the lesion and peripheral white scales, polymorphic vessels, with a linear and loop appearance, and a milky-red area.^6^ The arboriform vessels in NA make basal cell carcinoma an important dermoscopic differential diagnosis.

In the present report, in addition to the previously described findings, the authors observed the presence of shiny white structures and yellow dots on dermoscopy. Yellow dots similar to the ones seen on dermoscopy in alopecia areata and androgenetic alopecia, represent the ostia of hair follicles, which were also observed on histopathology, although NA classically occurs with cicatricial alopecia. It is possible that, in the early stages of the disease, hair follicles are still preserved.

Regarding cutaneous metastases in other anatomical regions, there are also few reports on their dermoscopic findings. Chernoff et al. suggested that the presence of vascular structures on dermoscopy of nodular lesions in cancer patients should call attention to the possibility of cutaneous metastasis in the differential diagnosis.^7^

Dermoscopy is a complementary and non-invasive examination that has been used for the diagnosis and management of alopecia in general. The clinical aspects of NA may resemble those of other diseases that present circumscribed alopecia. The knowledge of the dermoscopic structures of NA can help in the differential diagnosis of scalp dermatoses. Moreover, NA may be the first clinical manifestation of internal malignancy, making the diagnosis highly relevant for the patient.

Diagnostic suspicion of NA may also arise due to the absence of classic dermoscopic criteria of other scalp dermatoses in an area of alopecia or the presence of structures found in cutaneous metastases, such as arboriform vessels, telangiectasias within milky-red areas and polymorphic vessels.

## Financial support

None declared.

## Authors' contributions

Bruno Simão dos Santos: Intellectual participation in the propaedeutic and/or therapeutic conduct of the studied cases; design and planning of the study; drafting and editing of the manuscript; critical review of the manuscript; approval of the final version of the manuscript.

Eduardo César Diniz Macêdo: Drafting and editing of the manuscript; critical review of the literature.

Bruna Nascimento Arruda Scabello: Drafting and editing of the manuscript; critical review of the literature.

Patrícia Porto de Oliveira Grossi: Drafting and editing of the manuscript; critical review of the literature.

Neusa Yuriko Sakai Valente: Intellectual participation in the propaedeutic and/or therapeutic conduct of the studied cases; critical review of the manuscript; approval of the final version of the manuscript.

## Conflicts of interest

None declared.

## References

[1] Flanagan K.E., Burns L.J., Pathoulas J.T., Walker C.J., Pupo Wiss I., Cornejo K.M. (2021). Primary alopecia neoplastica: a novel case report and literature review. Skin Appendage Disord..

[2] Paolino G., Pampena R., Grassi S., Mercuri S.R., Cardone M., Corsetti P. (2019). Alopecia neoplastica as a sign of visceral malignancies: a systematic review. J Eur Acad Dermatol Venereol..

[3] Scheinfeld N. (2006). Review of scalp alopecia due to a clinically unapparent or minimally apparent neoplasm (SACUMAN). Acta Derm Venereol..

[4] Ferraro A., Argeiro A.L., Marques A.S., Dias Junior L.B., Antunes A.M. (1986). Metastatic alopecia of breast carcinoma (neoplasic alopecia). An Bras Dermatol..

[5] Vezzoni R., Toffoli L., Conforti C., Dri A., Retrosi C., di Meo N. (2021). Breast cancer-related neoplastic alopecia: a case report and review of the literature. Skin Appendage Disord..

[6] Çetinarslan T., Ermertcan A.T., Temiz P., Evrenos M.K., Müezzinoğlu T. (2020). Dermoscopy of scalp cutaneous metastasis of sarcomatoid renal cell carcinoma. Dermatol Ther..

[7] Chernoff K.A., Marghoob A.A., Lacouture M.E., Deng L., Busam K.J., Myskowski P.L. (2014). Dermoscopic findings in cutaneous metastases. JAMA Dermatol..

